# Abrogated Thioredoxin System Causes Increased Sensitivity to TNF-α-Induced Apoptosis via Enrichment of p-ERK 1/2 in the Nucleus

**DOI:** 10.1371/journal.pone.0071427

**Published:** 2013-09-06

**Authors:** Min-Hyuk Yoo, Bradley A. Carlson, Vadim N. Gladyshev, Dolph L. Hatfield

**Affiliations:** 1 Molecular Biology of Selenium Section, Laboratory of Cancer Prevention, Center for Cancer Research, National Institutes of Health, Bethesda, Maryland, United States of America; 2 Division of Genetics, Department of Medicine, Brigham and Women’s Hospital and Harvard Medical School, Boston, Massachusetts, United States of America; Florida International University, United States of America

## Abstract

Thioredoxin (Trx) and thioredoxin reductase 1 (TR1) are among the major redox regulators in mammalian cells and have a wide variety of roles, including removal of intracellular reactive oxygen species (ROS) and prevention of cell death. Tumor necrosis factor-α (TNF-α) induces cancer cell death. Although ROS have been proposed to participate in this process, the role of the thioredoxin system in TNF-α stimulated cell death remains unclear. We investigated the possibility that the thioredoxin system protects against TNF-α-induced cancer cell death by examining whether TR1/Trx1 status controls TNF-α-induced apoptosis in EMT6 murine breast cancer cells. TR1-deficient cells were more sensitive to TNF-α than control cells. Increased sensitivity to TNF-α was most pronounced in Trx1-deficient cells. TNF-α-induced nuclear localization of phosphorylated ERK 1/2 (p-ERK 1/2) correlated with increased apoptosis in TR1- and Trx1-deficient cells, suggesting a pro-apoptotic role for nuclear p-ERK 1/2 in TNF-α-induced apoptosis. In addition, phosphoinositide 3-kinase (PI3K) inhibition dramatically reduced TNF-α-stimulated apoptosis and nuclear localization of p-ERK 1/2. In contrast, inhibition of ROS, MEK, JNK, or p38 did not significantly alter p-ERK 1/2 localization or apoptosis in TR1- and Trx1-deficient cells compared to control cells. Further, NF-κB p65 localization was not changed in TR1- and Trx1-deficient cells in response to TNF-α relative to control cells. Our data suggest that the thioredoxin system plays a critical role in protecting against TNF-α-induced apoptosis by regulating the levels of nuclear p-ERK 1/2 in a PI3K-dependent manner.

## Introduction

The thioredoxin system is one of the most important cellular redox regulatory systems. Thioredoxin (Trx) and thioredoxin reductase 1 (TR1) are the principal components of this system. The thioredoxin system regulates the redox state of protein thiols and controls many cellular processes, including proliferation, defense against oxidative stress, and apoptosis [1–3]. Trx was first identified as a reductant for ribonucleotide reductase and a regulator of DNA synthesis [4,5]. However, Trx is now known to regulate many proteins in a variety of pathways. The molecular and biological targets of Trx include methionine sulfoxide reductase, which is involved in protein repair [6,7], nuclear factor-kappa B (NF-κB) and apoptosis signal-regulating kinase 1 (ASK1), which regulate apoptosis [8,9], and peroxiredoxin, which regulates levels of reactive oxygen species (ROS) [10].

Mammalian TR1 is a selenium-containing protein that has selenocysteine (Sec), the 21st amino acid, at its catalytic site. Sec is essential for the activity of TR1 [11,12]. TR1 is primarily known for its ability to catalyze the transfer of reducing potentials to thioredoxin from NADPH. However, TR1 has additional substrates, including selenocompounds, ascorbate, lipoate, and oxidized lipids. Thus, TR1 may regulate multiple cellular processes in addition to reducing Trx and, through function, regulate the cellular redox status [13–15]. Importantly, TR1 has been shown to be over-expressed in many human tumors and cancer cell lines. Numerous inhibitors of TR1 have been reported to impede tumor growth, suggesting that this selenoprotein may be a target for cancer therapy [16,17]. Indeed, knockdown of TR1 with small interfering RNA (siRNA) technology reversed many cancer phenotypes, providing further evidence that this antioxidant enzyme plays an important role in the progression and/or maintenance of cancer [18,19]. Cancer cells are also known to be more sensitive to selenite than normal cells. It was recently reported that TR1-deficient cancer cells were far more sensitive to selenite than the corresponding TR1-expressing cells [20]. These studies suggest that TR1 function may protect cells from extracellular stress. These data also uncovered a new role for TR1 in cancer, which is compensated for by the glutathione system and independent from its role in Trx1 reduction [21].

Extracellular signal-regulated kinases 1/2 (ERK 1/2) are part of one of the mitogen-activated protein kinase (MAPK) cascades, which regulate numerous cellular processes [22]. Exogenous stimulation of transmembrane receptors induces ERK signaling via molecules such as Raf and MEK. ERK signaling promotes multiple biological effects, such as proliferation, differentiation, survival, apoptosis, and morphology determination. It is not clear, however, how the ERK cascade discriminates between different stimuli or how activation of a single ERK cascade leads to different biological consequences. Differences in the intensity, duration, and cellular localization of ERK signaling may determine downstream signaling and biological effects. ERK 1/2 are localized predominantly in the cytoplasm of resting cells and are translocated to different cellular compartments, such as the nucleus, mitochondria, and endosomes upon stimulation [22,23]. Nuclear ERK 1/2 phosphorylate transcription factors that mediate a variety of physiological processes, including chromatin reorganization [24,25]. In addition, activated ERK 1/2 interact with proteins, such as β-arrestin and the p14-MP1 complex, and migrate to early or late endosomes. ERK1/2 regulate the trafficking of endosomes and internalized receptors [26,27]. ERK 1/2 were also found to localize to mitochondria and regulate processes including cell survival, apoptosis, and steroid synthesis [28,29].

To elucidate the role of TR1 and Trx1 in tumor necrosis factor (TNF)-α-induced apoptosis in cancer cells, we examined the sensitivity of TR1- or Trx1-deficient breast cancer cells to TNF-α. TR1 deficiency sensitized cells to TNF-α, and Trx1 deficiency resulted in an extremely high sensitivity to TNF-α. Levels of nuclear phospho-ERK 1/2 (p-ERK 1/2) were induced by TNF-α. The most significant increases in nuclear localization of p-ERK 1/2 were observed in cells that lacked TR1 and Trx1. In contrast, PI3K inhibition diminished levels of nuclear p-ERK 1/2 and rescued cells from TNF-α-induced apoptosis. Thus, abrogation of the thioredoxin system increased nuclear localization of p-ERK 1/2 and significantly increased apoptosis in response to TNF-α stimulation.

## Materials and Methods

### Materials

Recombinant mouse TNF-α, propidium iodide (PI), NuPage 4-12% Bis-Tris gels, polyvinylidene difluoride (PVDF) membrane, hygromycin B, Lipofectamine 2000, Waymouth’s MB 752/1 medium, antibiotic-antimycotic solution, and fetal bovine serum (FBS) were obtained from Invitrogen Life Technologies. PD98059, SB203580, JNK inhibitor II, and LY294002 were purchased from EMD Millipore Bioscience. The siRNA vector pSilencer 2.1-U6 Hygro was from Ambion, Inc. Antibodies against cleaved caspase-3 and phosphorylated and total ERK 1/2, p38, and JNK 1/2 were obtained from Cell Signaling Technology, Inc. The antibody against TR1 was from Epitomics. An anti-Trx1 antibody was from Abcam, Inc. The anti-p65 antibody was from Santa Cruz Biotechnology, Inc. Qproteome Mitochondria Isolation kit was purchased from QIAGEN. The BCA protein assay reagent and SuperSignal West Dura Extended Duration Substrate were from Thermo Fisher Scientific Inc. Protease inhibitor and phosphatase inhibitor cocktails were from Roche Diagnostics.

### Stable knockdown of TR1 and Trx1 in EMT6 cells and cell viability assay

The mouse mammary carcinoma cell line, EMT6, was obtained from the American Type Culture Collection. Cells were grown in Waymouth’s MB 752/1 media supplemented with 15% FBS and an antibiotic-antimycotic solution in a humidified incubator at 37^°^C with 5% CO_2_. Cells were stably transfected with pU6m3 (control), siTR1 (TR1 knockdown), and siTrx1 (Trx1 knockdown) constructs and were designated EMT/pU6m3, EMT/siTR1, and EMT/siTrx1, respectively, as previously described [20]. To measure sensitivity to TNF-α treatment, 1.0×10^6^ cells/dish were seeded onto 60-mm dishes and incubated overnight. Cells were left untreated or treated with TNF-α (10 ng/ml) for 48 h and harvested after washing with phosphate-buffered saline (PBS). Cell viability was measured by the trypan blue exclusion method. A Student’s t test was used to calculate the statistical significance of the experimental data.

### Preparation of nuclear, cytosolic, and mitochondrial extracts and western blot analysis

Cells were seeded onto a cell culture dish (1.0×10^6^/60-mm dish) and incubated overnight before treating with TNF-α (10 ng/ml). Cells were incubated for 10, 30, or 60 min and harvested after washing twice with PBS. To examine the effects of MAPK and PI3K inhibitors on the cellular localization of p-ERK 1/2, cells were treated with ERK inhibitor (PD98059, 10 μM), p38 inhibitor (SB203580, 5 μM), JNK inhibitor II (10 μM), PI3K inhibitor (LY294002, 5 μM), or buffer for 1 h and treated with TNF-α for 10 min. Cells were harvested after washing twice with PBS. All western blots were carried out in duplicate and representative data are shown in the figures.

**Figure 1:**
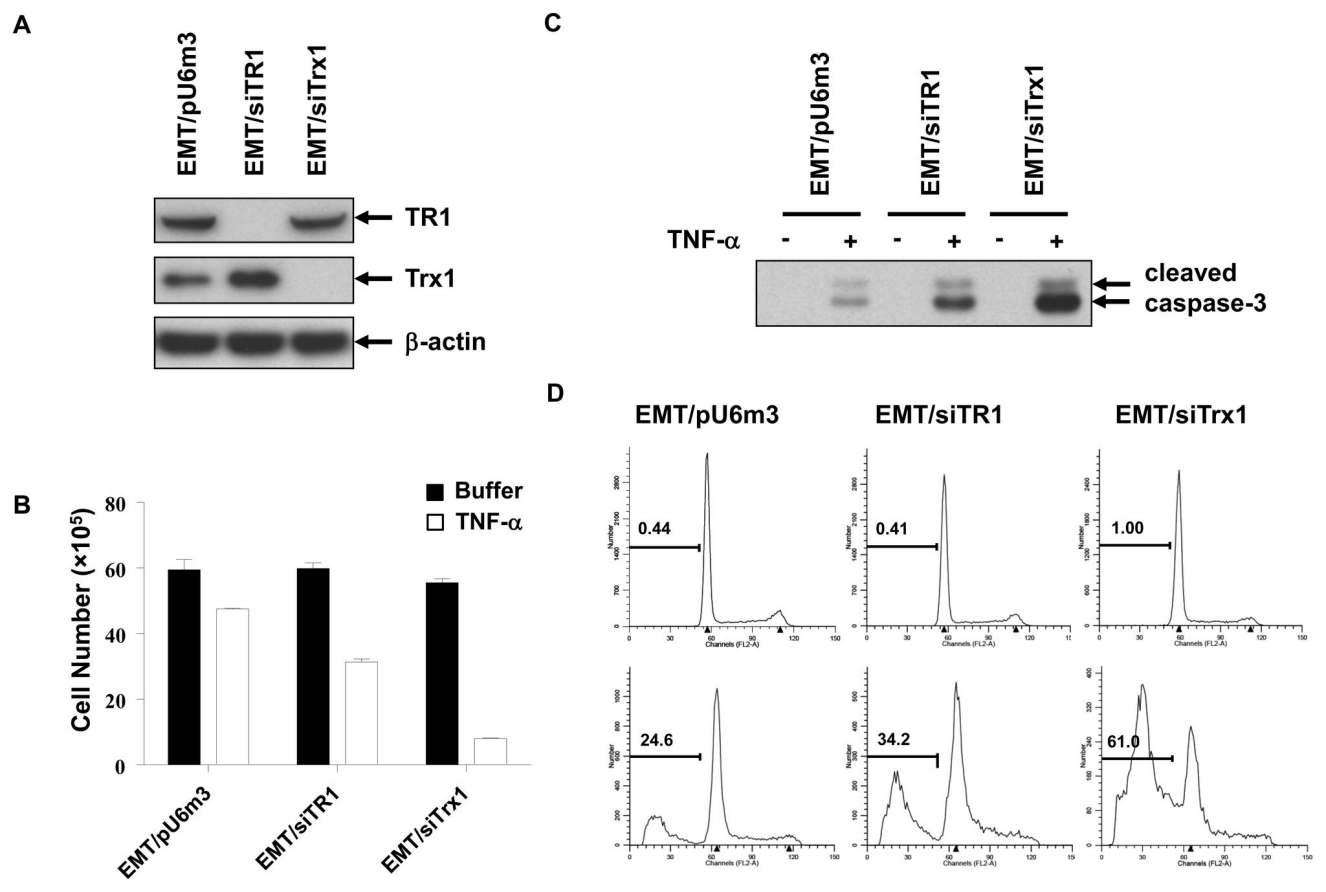
Knockdown of TR1 or Trx1 increases TNF-α-induced apoptosis in EMT6 cells. (A) EMT6 cells were stably transfected with pU6m3 (control), siTR1, or siTrx1 vectors, and expression levels of TR1 and Trx1 were analyzed by western blotting. β-Actin was examined as a loading control. (B) The viabilities of EMT/pU6m3, EMT/siTR1, and EMT/siTrx1 after TNF-α treatment (10 ng/ml) for 48 h were measured by staining with trypan blue and counting live cells. Darkened bars indicate untreated cells, and clear bars indicate TNF-α -treated cells. Values show the numbers of viable cells and are the means ±S.D (p<0.01). (C) EMT/pU6m3, EMT/siTR1, and EMT/siTrx1 cells were treated or untreated with TNF-α, and the level of cleaved capase-3 was detected in cell extracts by western blotting. (D) Cells were untreated or treated with TNF-α. DNA was stained with PI, and the DNA content was measured by flow cytometry. The percentages of apoptotic cells are shown in the graphs. The upper graphs show untreated cells, and the lower graphs show treated cells. Experimental details for each study are given in the Materials and Methods.

**Figure 2:**
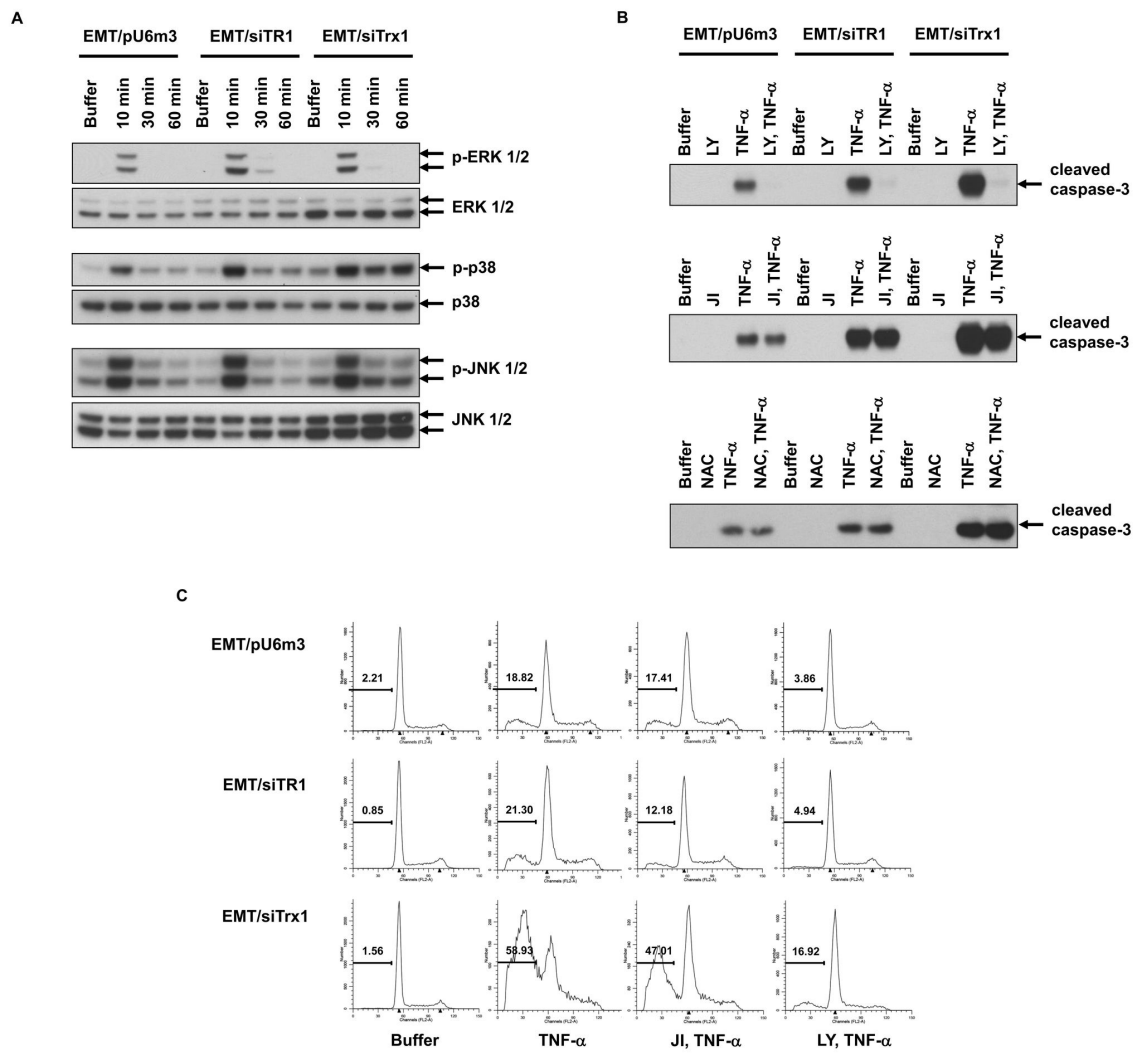
PI3K inhibition blocks TNF-α-induced apoptosis in EMT/pU6m3, EMT/siTR1, and EMT/siTrx1 cells. (A) Cells were untreated or treated with TNF-α (10 ng/ml) for the indicated time intervals. Phosphorylated (upper panels) and total ERK 1/2, p38, and JNK 1/2 (lower panels) were measured by western blotting. (B) Cells were pre-treated with PI3K inhibitor, LY294002 (5 µM, designated LY), JNK inhibitor II (10 μM, designated JI), NAC (0.5 mM), or buffer and treated with TNF-α. Cleaved caspase-3 was measured by western blotting. (C) Cells were pre-treated with buffer, JNK inhibitor II (JI) or LY294002 (LY) and then treated with TNF-α. The percentages of apoptotic cells were measured by flow cytometry and are shown in the graphs. Experimental details for each study are given in the Materials and Methods. Experiments were carried out in duplicate and representative data are shown.

**Figure 3:**
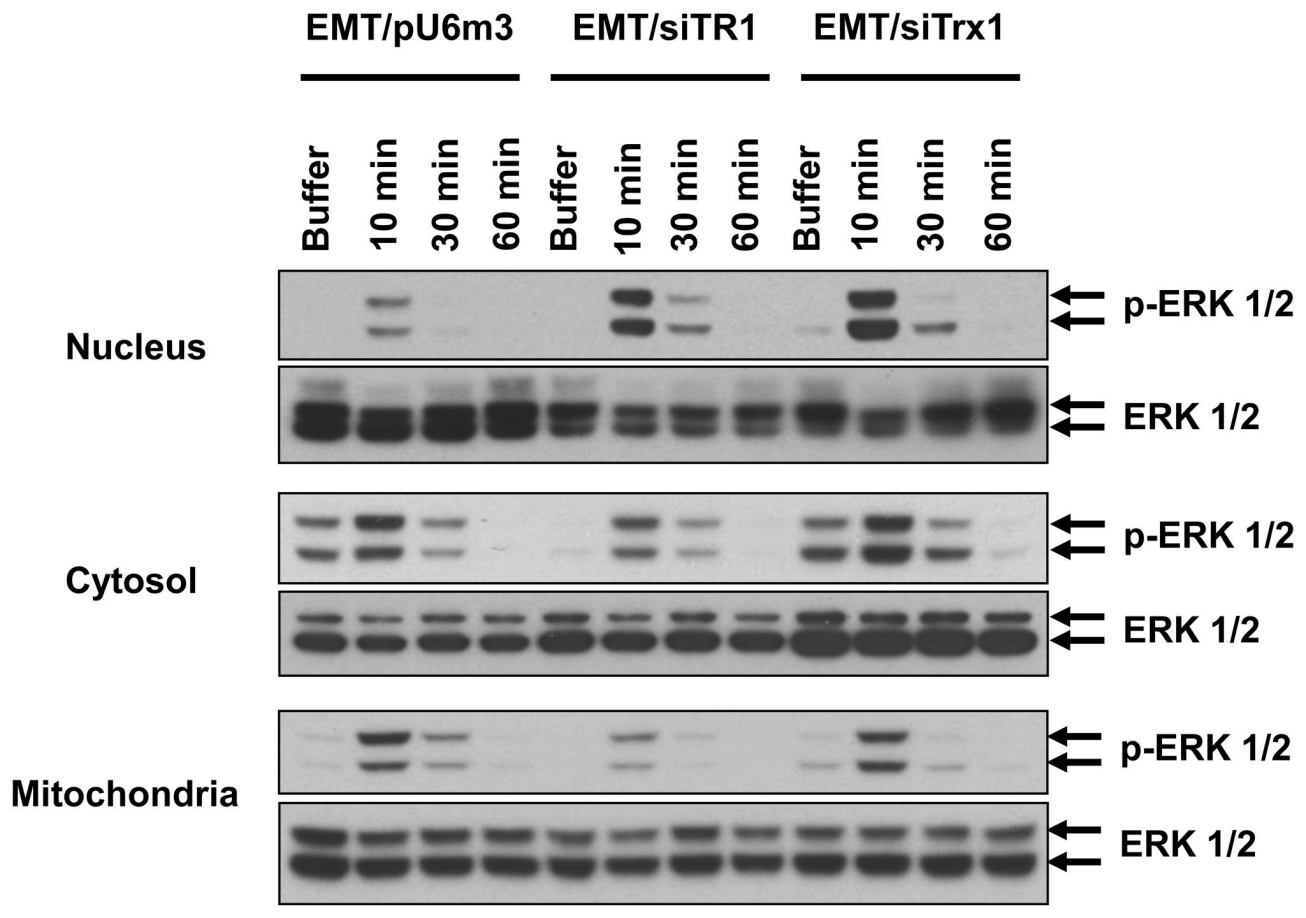
Phosphorylated ERK 1/2 is localized to the nucleus after TNF-α treatment. EMT/pU6m3, EMT/siTR1, and EMT/siTrx1 cells were exposed to buffer or TNF-α for the indicated time intervals. Cytosolic, nuclear, and mitochondrial protein extracts were western blotted for phosphorylated and total ERK 1/2 and are shown in the upper, middle, and lower panels, respectively.

**Figure 4:**
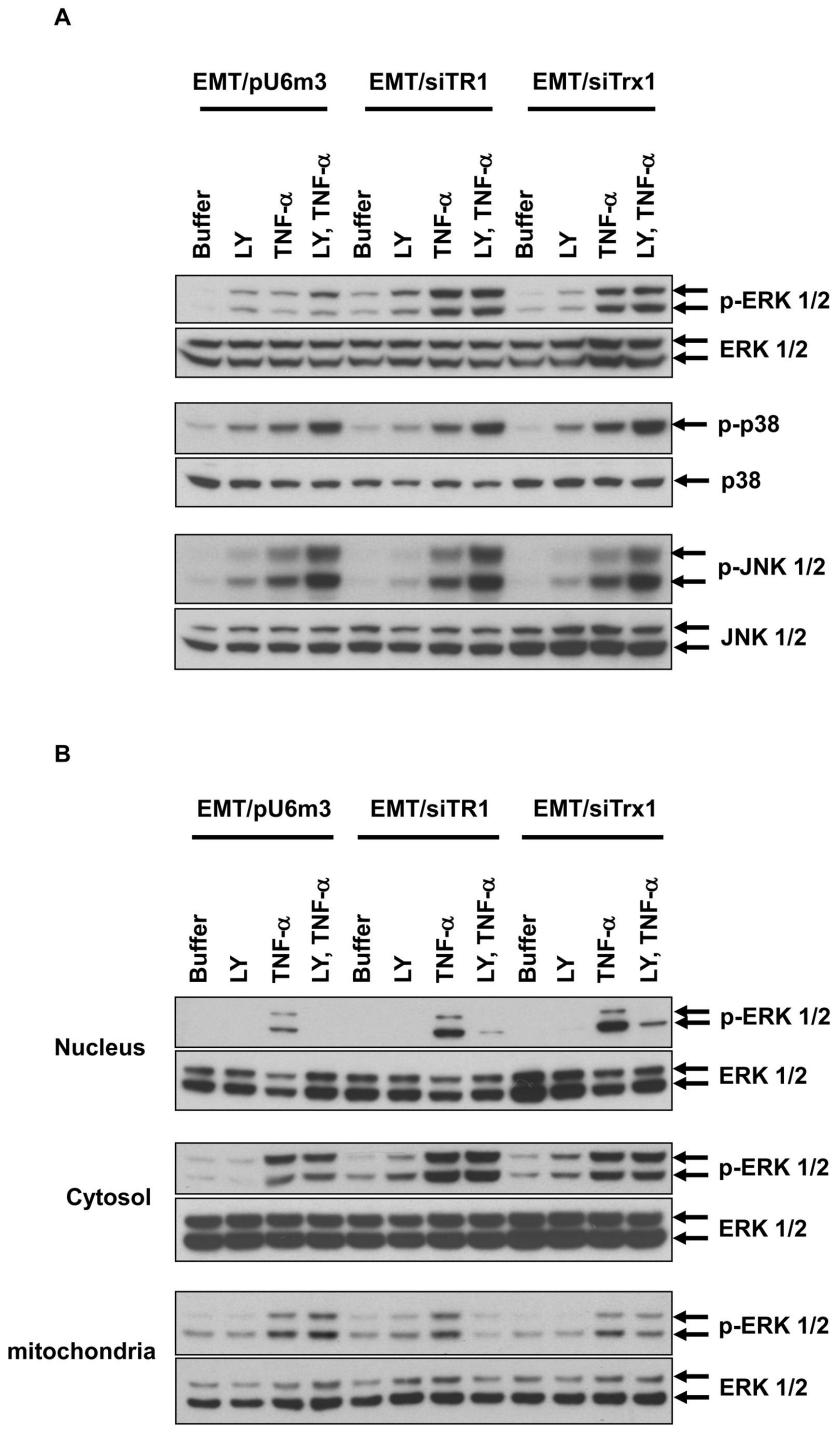
PI3K inhibition blocks TNF-α-induced nuclear re-localization of p-ERK 1/2. EMT/pU6m3, EMT/siTR1, and EMT/siTrx1 cells were initially treated or untreated with LY294002 (5 μM, designated LY) for 1 h and treated or untreated with TNF-α. (A) Phosphorylated and total ERK 1/2, p38, and JNK were measured in total cellular lysates by western blotting (B) Phosphorylated and total ERK 1/2 in nuclear (upper), cytosolic (middle), and mitochondrial (lower) fractions were measured by western blotting.

Whole-cell protein samples were prepared with lysis buffer (10 mM HEPES, pH 7.9, 10 mM KCl, 0.1 mM EGTA, 0.1 mM EDTA, 1 mM dithiothreitol (DTT), protease inhibitor cocktail, and phosphatase inhibitor cocktail) as described elsewhere [18]. Nuclear extracts were prepared as described previously [30]. In brief, harvested cells were incubated on ice for 10 min, ruptured by vortexing for 10 sec after adding Igepal (0.3% final concentration), and centrifuged at 13,000 × *g* for 1 min at 4°C. Pellets, which contained nuclei, were suspended with nuclear extraction buffer (20 mM HEPES, pH 7.9, 0.5 mM EGTA, 0.5 mM EDTA, 420 mM NaCl, 1 mM DTT, protease inhibitors, and phosphatase inhibitors), incubated on ice for 30 min with intermittent vortexing, and centrifuged at 13,000 × *g* for 15 min at 4°C. Mitochondrial extracts were prepared with Qproteome mitochondria isolation kit following the manufacturer’s instructions. Protein concentrations were quantified with BCA protein assay reagent. Protein samples were electrophoresed on NuPAGE 4-12% Bis-Tris gels, and the separated protein samples were transferred to a PVDF membrane. The membrane was incubated with a primary antibody and washed with TBS-T. The membrane was incubated with a horseradish peroxidase-conjugated secondary antibody, exposed to SuperSignal West Dura Extended Duration Substrate, and visualized on X-ray film. The corresponding uncropped figures of western blots presented in the text (Figures 1A, 1C, 2A, 2B, 3, 4A and 4B) are shown as Figure S4.

### Quantification of apoptotic cells by flow cytometry

Cells (1.5×10^6^/60-mm dish) were seeded onto culture dishes and incubated overnight. Cells were pre-treated with ERK inhibitor (PD98059, 10 μM), p38 inhibitor (SB203580, 5 μM), JNK inhibitor II (10 μM), PI3K inhibitor (LY294002, 5 μM), or buffer for 1 h and treated with TNF-α for 24 h. Cells were harvested and fixed with cold 70% ethanol overnight at 4°C. Fixed cells were centrifuged, re-suspended in PBS containing RNase A, and incubated for 30 min at 37°C. Cells were stained overnight in the dark with PI (50 μg/ml) and analyzed by flow cytometry on a FACS Calibur 2 Sorter (Becton Dickinson). The apoptotic cell populations were quantified by analyzing data with FlowJo (Tree Star, Inc.).

## Results

### Generation of EMT6 cells with stable knockdown of TR1 or Trx1

We stably knocked down TR1 or Trx1 in EMT6 cells with siRNA constructs that have previously been shown to be efficient in decreasing TR1 or Trx1 expression (Figure 1A) [20]. Expression of TR1 or Trx1 was down-regulated by more than 90% in stable EMT/siTR1 and EMT/siTrx1 cells, respectively, in comparison to control cells expressing EMT/pU6m3 empty vector. Interestingly, an increase in Trx1 expression was observed upon knockdown of TR1, whereas Trx1 knockdown did not cause a significant change in TR1 levels.

### TNF-α-induced apoptosis is increased in TR1- or Trx1-deficient cells

Because TNF-α induces cell death [31], the relative sensitivities of the control, TR1-deficient, and Trx1-deficient cells to TNF-α were examined (Figure 1B). In comparison to untreated cells, approximately 80%, 50%, and 15% of the control, TR1-deficient, and Trx1-deficient cells, respectively, survived. Although the TR1-deficient and Trx1-deficient cells both showed increased sensitivity to TNF-α versus control cells, Trx1-deficient cells were the most sensitive.

To determine if the increased sensitivities of TR1-deficient or Trx1-deficient cells to TNF-α were a reflection of increased apoptosis, we examined activation of caspase-3, which is a major marker of apoptosis. Full length caspase-3 must be cleaved to become an active protease that mediates apoptosis [32,33]. Caspase-3 activation by TNF-α was examined by western blot analysis in the stable cell lines (Figure 1C). Caspase-3 was activated in each of the cell lines. However, the levels of cleaved caspase-3 were higher in TR1-deficient cells relative to control cells and were even more pronounced in Trx1-deficient cells.

Apoptosis was also examined after TNF-α treatment in each cell line by analyzing cellular DNA content by flow cytometry (Figure 1D). The percentages of apoptotic cells were approximately 25%, 34%, and 60% for control, TR1-deficient, and Trx1-deficient cells, respectively. The relative levels of caspase-3 correlated with the levels of apoptosis. These data suggested that reduced viability (Figure 1B) was due to induction of apoptosis in response to TNF-α treatment.

### Inhibition of PI3K with LY294002 rescues TNF-α-induced apoptosis in TR1- and Trx1-deficient cells

TNF-α activates NF-κB and promotes nuclear translocation of p65, which suppresses TNF-α-induced apoptosis [34] and activates the MAPK pathways inducing various biological process such as inflammation, proliferation and cell death [35,36]. To assess the involvement of increasingly higher levels of apoptosis in TR1- and Trx1-deficient cells than control cells induced by TNF-α, the amount of p65 imported into the nucleus following TNF-α treatment in the three cell lines was initially examined (Figure S1A). p65 levels increased substantially in the nucleus at 10 min, appeared to decline at 30 min and were virtually absent at 60 min in each cell line. Simultaneously, the levels in the cytoplasm appeared to decrease at 10 min and then increase by 60 min in an approximate balanced manner between the nucleus and cytoplasm over the course of the experiment. But TR1 or Trx1 deficiencies did not lead to any significant change in the nuclear translocation of p65 by TNF-α treatment compared to the corresponding control.

Activation of the MAPK proteins, ERK 1/2, p38 and JNK 1/2, which occurs upon their phosphorylation, was next examined (Figure 2A). These three MAPKs were strongly phosphorylated at 10 min and quickly deactivated in each cell line with the exception of phosphorylated p38 that was maintained up to 60 min in Trx1-deficient cells. The levels of ERK 1/2 and JNK 1/2 prior to phosphorylation were substantially higher in Trx1-deficient cells compared to the other two cell lines, and the possible reason for this enrichment was unclear.

Because the PI3K pathway is known to affect ERK 1/2 signaling cascade [37], the involvement of MAPK and PI3K pathways with the enhanced apoptosis caused by TR1- or Trx1-deficient cells was further investigated. The effects of an ERK 1/2 inhibitor, PD98059, a JNK 1/2 inhibitor, JNK inhibitor II, a p38 inhibitor, SB203580, and a PI3K inhibitor, LY294002, on TNF-α-induced caspase-3 activation were examined. Inhibition of ERK 1/2 or p38 increased caspase-3 activation (Figure S1B). In contrast, LY294002 almost completely blocked caspase-3 activation in all three cell lines (Figure 2B, upper panel). JNK inhibition did not significantly affect caspase-3 activation (Figure 2B, middle panel). Because the thioredoxin system is primarily known as a regulator of cellular redox status, the involvement of ROS in TNF-α-induced apoptosis was examined (Figure 2B, lower panel). Cells were treated with an ROS inhibitor, NAC (N-acetyl cysteine), and caspase-3 activation was assessed by western blotting. NAC did not alter caspase-3 activation, suggesting that ROS did not play a major role in mediating the increased apoptosis of TR1- and Trx1-deficient cells.

To confirm the data regarding kinase inhibition and caspase-3 activation, we examined the effects of kinase inhibitors on apoptosis in TR1- and Trx1-deficient cells following TNF-α treatment. Inhibition of ERK 1/2 and p38, which increased caspase-3 activation, also resulted in a slight increase in apoptosis in control and TR1-deficient cells and virtually no change in Trx1-deficient cells (Figure S2). However, JNK inhibition, which did not affect caspase-3 activation, resulted in a slight rescue from apoptosis in TR1- and Trx1-deficient cells, Treatment with LY294002, which completely blocked the activation of caspase-3, dramatically rescued apoptosis in all three cell lines (Figure 2C, columns 3 and 4, respectively). A small percentage of LY294002-treated, TNF-α-stimulated Trx1-deficient cells were apoptotic.

### Knockdown of TR1 or Trx1 increases TNF-α-induced nuclear localization of p-ERK 1/2

ERK 1/2 is known to translocate to the nucleus and mitochondria upon activation by various stimuli [22,23]. The amounts of p-ERK 1/2 were measured in the nucleus, cytosol, and mitochondria to determine if localization of p-ERK 1/2 contributes to the enhanced sensitivity of TR1- and Trx1-deficient cells to TNF-α (Figure 3). Phosphorylation of ERK 1/2 was induced within 10 min of TNF-α treatment in all three cellular compartments similar to total p-ERK 1/2 (compare Figure 3 with Figure 2A, upper panel). Similar levels of cytosolic and mitochondrial p-ERK 1/2 were observed in control and Trx1-deficient cells, whereas TR1-deficient cells had the lowest level of mitochondrial p-ERK 1/2. Importantly, the amounts of p-ERK 1/2 in the nucleus were significantly higher in TR1-deficient cells and highest in Trx1-deficient cells, which suggested a correlation between levels of nuclear p-ERK 1/2 and apoptosis following TNF-α treatment.

### Inhibition of PI3K blocks TNF-α-induced nuclear localization of p-ERK 1/2

Because PI3K inhibition rescued cells from apoptosis (Figure 2B and 2C), the role of PI3K in the nuclear localization of p-ERK 1/2 was examined (Figure 4). The effects of LY294002 on ERK 1/2 phosphorylation in total cellular lysates (Figure 4A) was compared to p-ERK 1/2 in cellular compartments (Figure 4B). Inhibition of PI3K before TNF-α treatment did not cause a significant change in total p-ERK 1/2. However, LY294002 increased the levels of phosphorylated p38 and JNK 1/2 (Figure 4A). The amounts of p-ERK 1/2 in the cytosol and mitochondria were slightly affected by LY294002 treatment, whereas mitochondrial p-ERK 1/2 in TR1-deficient cells was diminished (Figure 4B, middle and lower panels). As expected, inhibition of PI3K prior to TNF-α treatment almost completely abolished the increased level of nuclear p-ERK 1/2 (Figure 4B, upper panel).

## Discussion

The thioredoxin system is an important molecular target for anti-cancer therapy. Increased expression levels of TR1 and Trx1 have been reported in various cancer models in association with poor prognosis, increased proliferation, and high tumor grade [38–40]. We previously reported that TR1 has a role in promoting or maintaining cancer. Knockdown of TR1 has been shown to reduce anchorage-independent growth, tumor growth, metastasis, and self-sufficient growth signaling in cancer cells [18,19]. These studies support inhibition of the thioredoxin system as a therapeutic strategy in cancer. However, the mechanistic basis by which the thioredoxin system inhibition suppresses cancer progression remains unclear.

In this study, we present data that suggest that loss of Trx1 or TR1 increases TNF-α-induced apoptosis in breast cancer cells by localizing phosphorylated ERK 1/2 to the nucleus in a PI3K-dependent manner. Stable knockdown of TR1 or Trx1 caused increased caspase-3 cleavage, reduced cell survival, and increased apoptosis in response to TNF-α relative to control cells. Increased apoptosis of thioredoxin system-defective cells was associated with significantly increased levels of nuclear p-ERK 1/2. In contrast, cytosolic and mitochondrial p-ERK 1/2 did not correlate with the increased sensitivity of TR1- and Trx1-deficient cells to TNF-α. These results suggest that cellular localization of p-ERK 1/2 is a critical determinant of whether cells with defective thioredoxin function will undergo apoptosis in response to particular stimuli, such as TNF-α.

Phosphorylation of ERK 1/2 is regulated in part by PI3K signaling [37]. Thus, we examined the effect of PI3K inhibition on the cellular localization of p-ERK 1/2. We found that PI3K inhibition prevented TNF-α-induced apoptosis in association with localization of p-ERK 1/2. Levels of nuclear p-ERK 1/2 were almost completely suppressed by PI3K inhibition, whereas cytosolic and mitochondrial p-ERK 1/2 levels were only slightly affected by LY294002. These observations suggested that PI3K signaling regulated TNF-α-induced apoptosis by controlling nuclear localization of p-ERK 1/2. The specificity of PI3K signaling involvement in this process was supported by data showing that inhibition of MEK or p38 MAPK did not significantly alter TNF-α-induced apoptosis in thioredoxin-defective cells relative to control cells. Further, although MAPK and NF-κB were strongly activated by TNF-α, there were not significant differences between TR1- or Trx1-deficient cells and control cells. The effects of MAPK inhibitors on caspase-3 activation and apoptosis did not correlate with the increased sensitivity of TR1- or Trx1-deficient cells. Importantly, however, inhibition of PI3K with LY294002 almost completely blocked caspase-3 activation in all of three cell lines and rescued virtually all of the control and TR1-deficient cells from apoptosis. In addition, ~65% of the apoptotic cells were recovered by inhibiting PI3K in Trx1-deficient cells. These latter observations suggested that PI3K plays a major role in regulating TNF-α-induced apoptosis. Trx1 deficiency also appeared to cause additional defects in these cells, resulting in greater sensitivity to TNF-α.

The specific down-regulation of nuclear p-ERK 1/2 subsequent to PI3K inhibition resulted in an almost complete recovery from apoptosis. These data suggest a pro-apoptotic role for nuclear p-ERK 1/2 and an anti-apoptotic role for p-ERK 1/2 localized in other cellular compartments. PI3K has been shown to affect the activity of ERK 1/2. Tropomyosin-1-induced microfilaments and RhoA were involved in nuclear localization of ERK 1/2 [41,42]. ASK1 is an example of kinase whose activity is controlled by interaction with Trx, wherein the binding is dependent on the redox status of Trx. Many other kinases and phosphatases have been reported to be regulated by Trx, but in most cases the mechanisms are poorly understood [2]. Casein kinase 2 also regulated the nuclear transport of ERK 1/2 by phosphorylating two Ser residues within a nuclear translocation signal [43]. Trx1 deficiency inhibited the activation and the nuclear localization of ERK 1/2 after epidermal growth factor (EGF) treatment, but the role of nuclear ERK 1/2 was not examined [44]. The function and mechanism of PI3K in the translocation of ERK 1/2 to specific cellular organelles requires further investigation. In contrast to nuclear p-ERK 1/2, total nuclear ERK 1/2 was not significantly altered by TNF-α treatment. Similarly, cytosolic and mitochondrial ERK 1/2 levels were unchanged in response to TNF-α. Thus, TNF-α-stimulated breast cancer cells that have lost TR1 or Trx1 showed specific localization of p-ERK 1/2 to the nucleus in association with apoptosis. Further investigation will establish the upstream pathways that activate PI3K signaling and additional mechanisms regulating ERK 1/2 translocation to specific cellular organelles in TR1- and Trx1-deficient cells.

Another important mechanism of TNF-α-induced apoptosis is the induction of ROS levels via NADPH oxidase (NOX) activation. Pre-treatment with NAC decreases ROS induction in response to TNF-α stimulation [45,46]. However, we found that pre-treatment of cells with NAC prior to TNF-α treatment did not affect caspase-3 activation, suggesting that ROS did not significantly contribute to TNF-α-induced apoptosis in EMT6/TR1- or Trx1-deficient cells. Activation of NF-κB is also known to suppress TNF-α-induced apoptosis [34]. However, p65 localization to the nucleus, which is an indication of activation, was not altered by MAPK or PI3K inhibition after TNF-α treatment (Figure S3). Thus, PI3K inhibition appeared to rescue TNF-α-induced apoptosis in an NF-κB-independent manner.

Our data further indicated that cells with Trx1 knockdown showed a more significant reduction in cell survival (15%) than TR1-deficient cells (50%). A similar trend was observed with caspase-3 activation and apoptosis. Previous studies have shown that TR1 knockdown led to a significantly higher level of selenium cytotoxicity in mouse DT cells in comparison to Trx1 loss in these cells [20]. The enhanced cytotoxicity of selenium in TR1-deficient cells was caused by increased production of glutathione, suggesting that TR1 regulates glutathione production and secretion in a Trx1-independent manner. Trx1-deficient breast cancer cells manifested a particularly high sensitivity to TNF-α-induced apoptosis. These results support a protective or anti-apoptotic role for the thioredoxin system. In addition, our data demonstrate dependence of TR1 on Trx1 in protecting cells against TNF-α-induced apoptosis. Trx regulates the activity of ASK1 which is one of the major apoptosis regulators [2]. Future studies on the involvement of ASK1 in PI3K signaling and regulation of TNF-α induced apoptosis by controlling nuclear p-ERK1/2 may provide further insights into the role of the thioredoxin system in apoptosis.

A potential limitation of the study was the use of a single murine breast cancer cell line. However, EMT6 cells are a well-established model system for studying molecular abnormalities in breast cancer. Further studies will need to be performed in human breast cancer cell lines. In particular, and due to the critical role of PI3K signaling in the apoptotic response of TR1- and Trx1-deficient cells, breast cancer cell lines that harbor constitutive activation of the PI3K signaling pathway will also need to be examined. A subset of human breast cancers are known to express *PIK3CA* hyper-activating mutations or loss of the *PTEN* phosphatase, resulting in constitutively activated PI3K [47,48]. Our data suggested that PI3K signaling mediates the heightened apoptotic response to TNF-α in cells with defective thioredoxin systems. Thus, one can hypothesize that inhibition of TR1, and especially Trx1, may be a highly effective strategy for inducing apoptosis in breast cancer cells that harbor endogenous hyper-activation of PI3K signaling.

In summary, the following novel findings were made with the cancer cell lines with stable knockdown of the thioredoxin system(1). Loss of thioredoxin function increased the apoptotic response of murine breast cancer cells to TNF-α(2). Levels of nuclear p-ERK 1/2 were increased in association with apoptosis in TNF-α-treated thioredoxin-defective cells(3). TNF-α-induced apoptosis and localization of nuclear p-ERK 1/2 were dependent upon PI3K signaling. Thus, abrogation of the thioredoxin system enhanced sensitivity to TNF-α through a PI3K-dependent mechanism involving localization of phosphorylated ERK 1/2 to the nucleus. Our results provide novel insights into the mechanisms by which inhibition of TR1 and Trx1 promotes apoptosis of cancer cells and strongly support thioredoxin system blockade as a novel therapeutic strategy for cancer.

## Supporting Information

Figure S1 **Nuclear translocation of p65 by TNF-α treatment and the effect of MAPK inhibitors on TNF-α-induced caspase-3 activation.** (A) EMT/pU6m3, EMT/siTR1, and EMT/siTrx1 cells were untreated or treated with TNF-α for the indicated time intervals. Cytosolic and nuclear extracts were prepared as stated in the Materials and Methods section. Expression of p65 was measured by western blotting. (B) Cells were pre-treated with PD98059 (PD) or SB203580 (SB) for 1 h and treated with TNF-α. Cleaved caspase-3 was examined after 24 h by western blotting.
(PDF)

Figure S2 **Effect of PD98059 and SB209580 on TNF-α-induced apoptosis.** EMT/pU6m3, EMT/siTR1, and EMT/siTrx1 cells were pre-treated with MEK inhibitor, PD98059 (10 μM, designated PD), and p38 inhibitor, SB203580 (5 μM, designated SB), for 1 h and incubated with TNF-α. The percentages of apoptotic cells were measured by examining DNA contents after staining with PI and are shown in the graphs. Experimental details are given in the Materials and Methods section.
(PDF)

Figure S3 **Effect of MAPKs and PI3K inhibitors on the nuclear translocation of p65 by TNF-α.** Cells were pre-treated with PD98059, SB203580, JNK inhibitor II, or LY294002 and treated with TNF-α. Nuclear extracts were western blotted for p65. Experimental details are given in the Materials and Methods section.
(PDF)

Figure S4 **Uncropped figures of western blots presented in the text (Figures 1A, 1C, 2A, 2B, 3, 4A and 4B).**
(PDF)
