# Supplementary figures and images for: Abrogated Thioredoxin System Causes Increased Sensitivity to TNF-α-Induced Apoptosis via Enrichment of p-ERK 1/2 in the Nucleus
Source: PLoS One. 2013 Sep 6;8(9):e71427. doi: 10.1371/journal.pone.0071427 (PMC3765418; doi:10.1371/journal.pone.0071427)

**A**

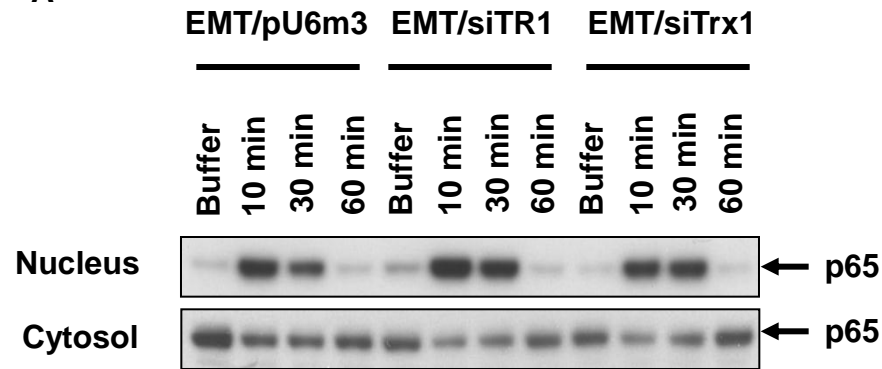

**B**

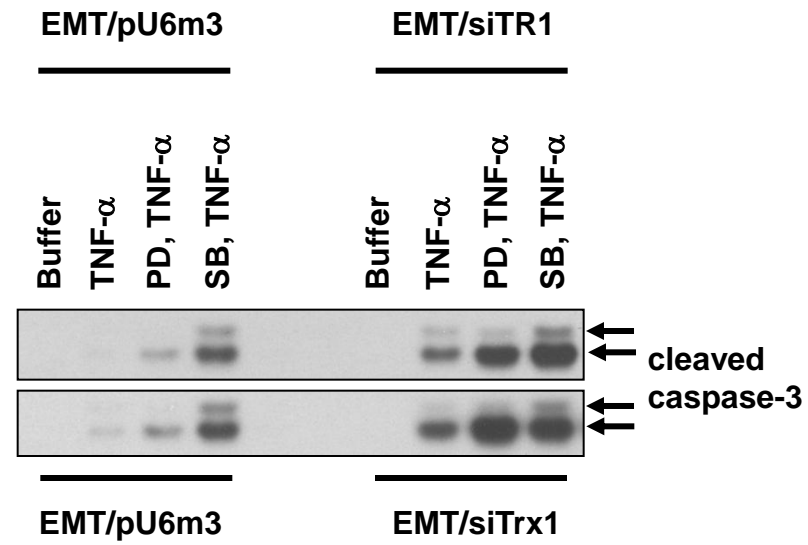

Supplement: Figure S1 — Nuclear translocation of p65 by TNF-α treatment and the effect of MAPK inhibitors on TNF-α-induced caspase-3 activation. (A) EMT/pU6m3, EMT/siTR1, and EMT/siTrx1 cells were untreated or treated with TNF-α for the indicated time intervals. Cytosolic and nuclear extracts were prepared as stated in the Materials and Methods section. Expression of p65 was measured by western blotting. (B) Cells were pre-treated with PD98059 (PD) or SB203580 (SB) for 1 h and treated with TNF-α. Cleaved caspase-3 was examined after 24 h by western blotting. (PDF) [file pone.0071427.s001.pdf]

**EMT/pU6m3**

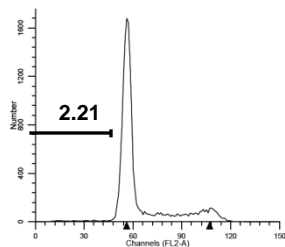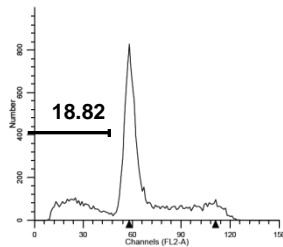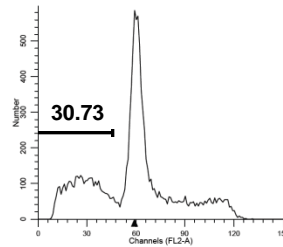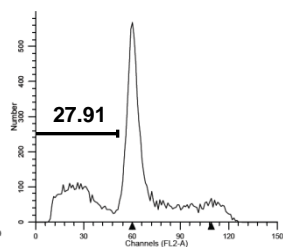

**EMT/siTR1**

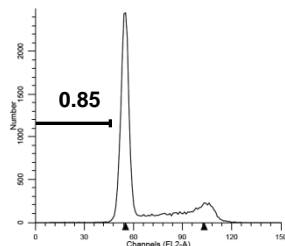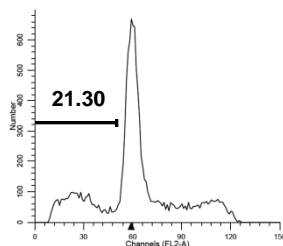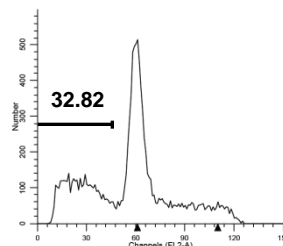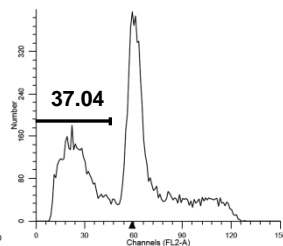

**EMT/siTrx1**

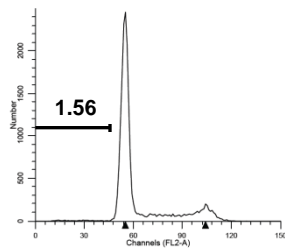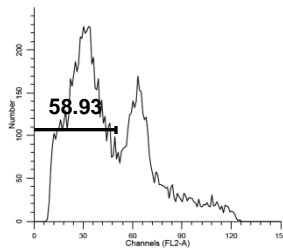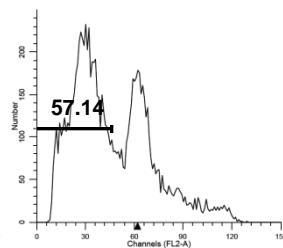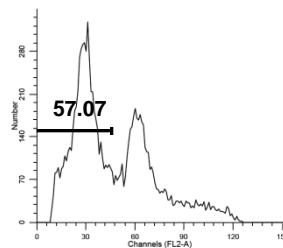

**Buffer**

**TNF- $\alpha$**

**PD, TNF- $\alpha$**

**SB, TNF- $\alpha$**

Supplement: Figure S2 — Effect of PD98059 and SB209580 on TNF-α-induced apoptosis. EMT/pU6m3, EMT/siTR1, and EMT/siTrx1 cells were pre-treated with MEK inhibitor, PD98059 (10 μM, designated PD), and p38 inhibitor, SB203580 (5 μM, designated SB), for 1 h and incubated with TNF-α. The percentages of apoptotic cells were measured by examining DNA contents after staining with PI and are shown in the graphs. Experimental details are given in the Materials and Methods section. (PDF) [file pone.0071427.s002.pdf]

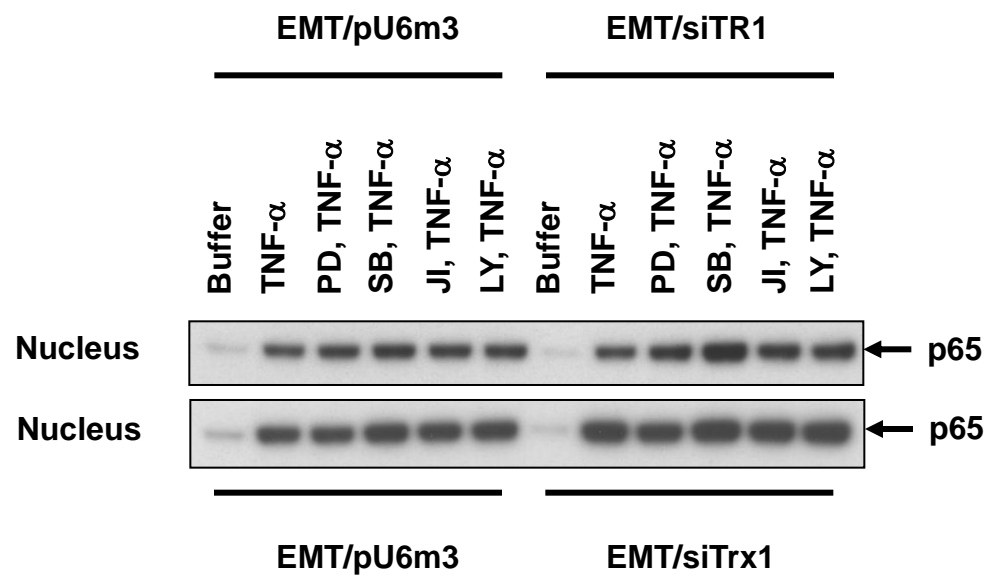

Supplement: Figure S3 — Effect of MAPKs and PI3K inhibitors on the nuclear translocation of p65 by TNF-α. Cells were pre-treated with PD98059, SB203580, JNK inhibitor II, or LY294002 and treated with TNF-α. Nuclear extracts were western blotted for p65. Experimental details are given in the Materials and Methods section. (PDF) [file pone.0071427.s003.pdf]
