# Supplementary material for: Abrogated Thioredoxin System Causes Increased Sensitivity to TNF-α-Induced Apoptosis via Enrichment of p-ERK 1/2 in the Nucleus
Source: PLoS One. 2013 Sep 6;8(9):e71427. doi: 10.1371/journal.pone.0071427 (PMC3765418; doi:10.1371/journal.pone.0071427)
Supplement: Figure S4 — Uncropped figures of western blots presented in the text (Figures 1A, 1C, 2A, 2B, 3, 4A and 4B). (PDF) [file pone.0071427.s004.pdf]

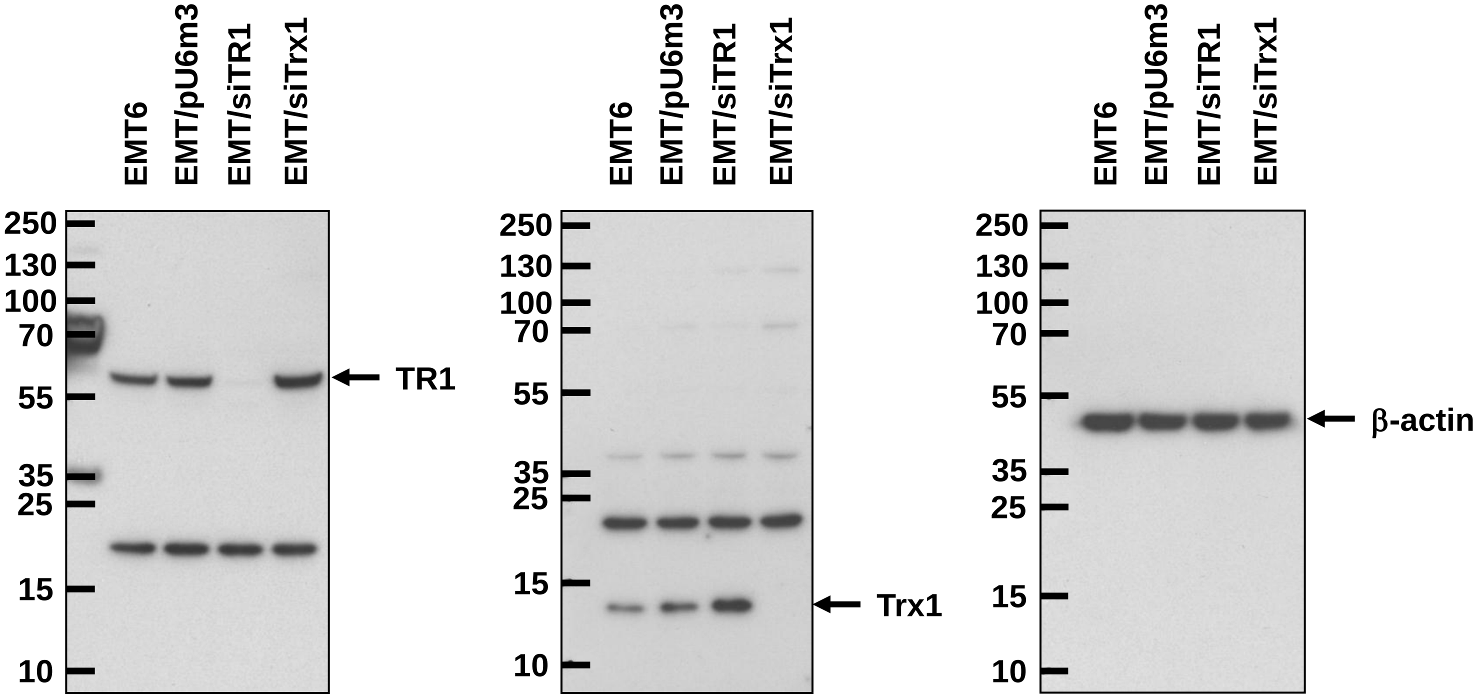
EMT6
EMT/pU6m3
EMT/siTR1
EMT/siTrx1
250
130
100
70
55
35
25
15
10
TR1
Trx1
β-actin

Figure 1A

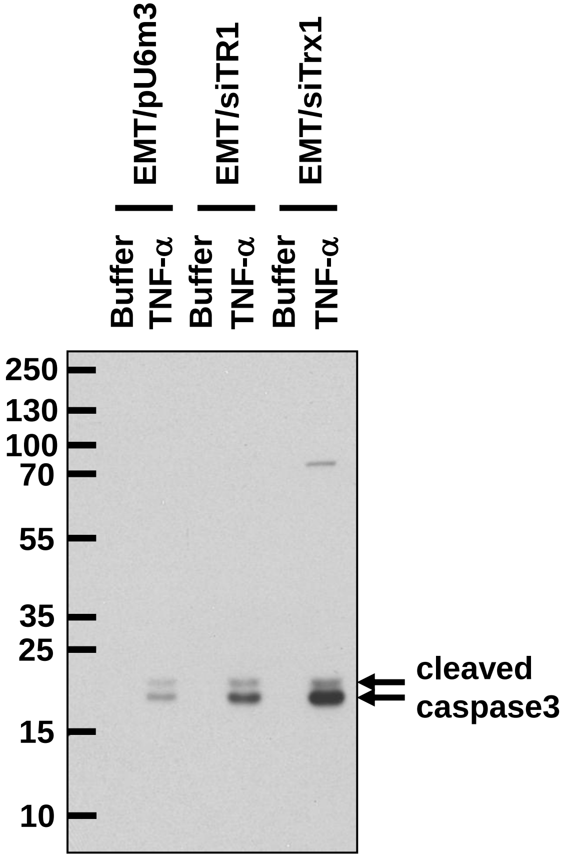
EMT/pU6m3
EMT/siTR1
EMT/siTrx1
Buffer
TNF-α
Buffer
TNF-α
Buffer
TNF-α
250
130
100
70
55
35
25
15
10
cleaved
caspase3

Figure 1C

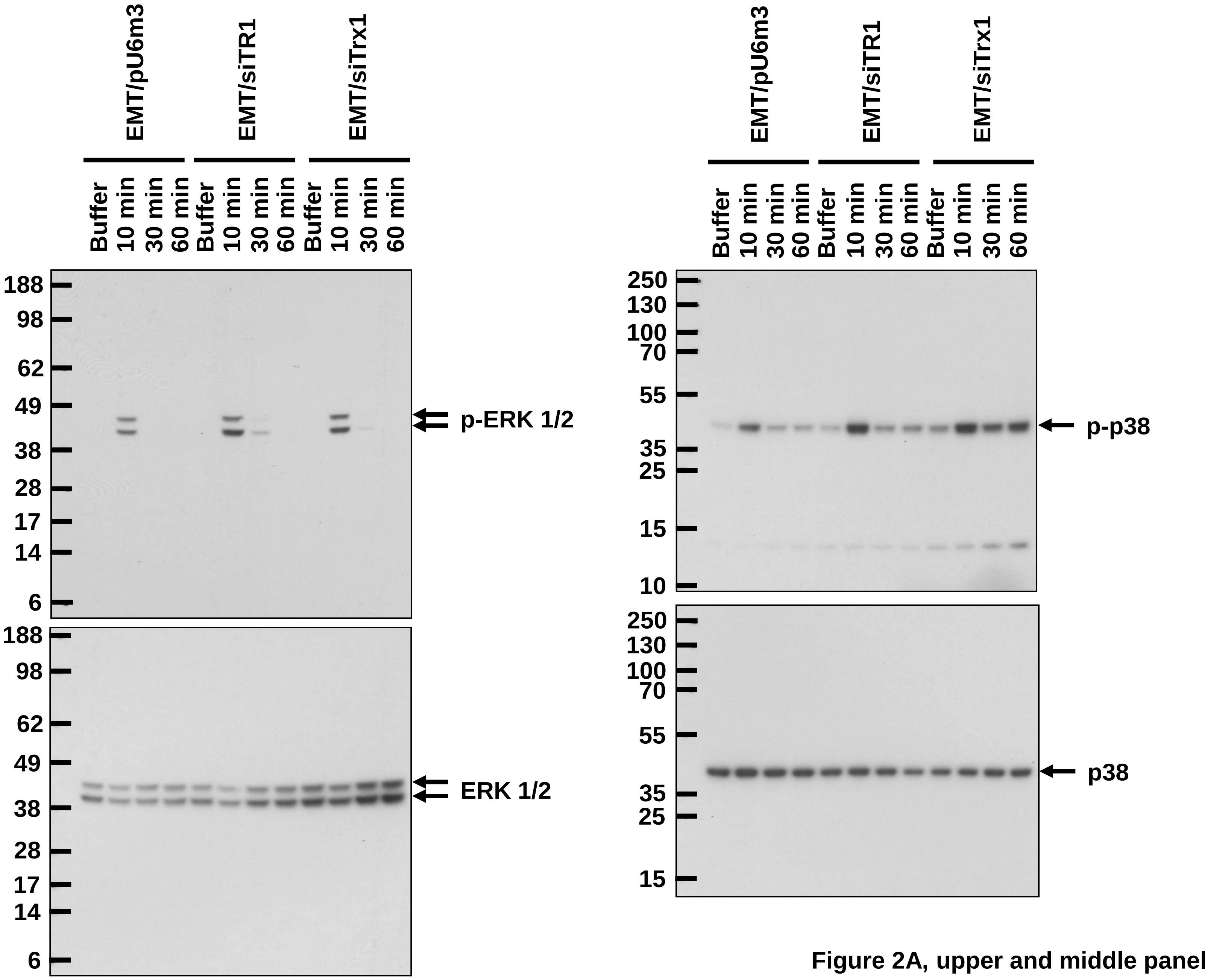

Figure 2A, upper and middle panel

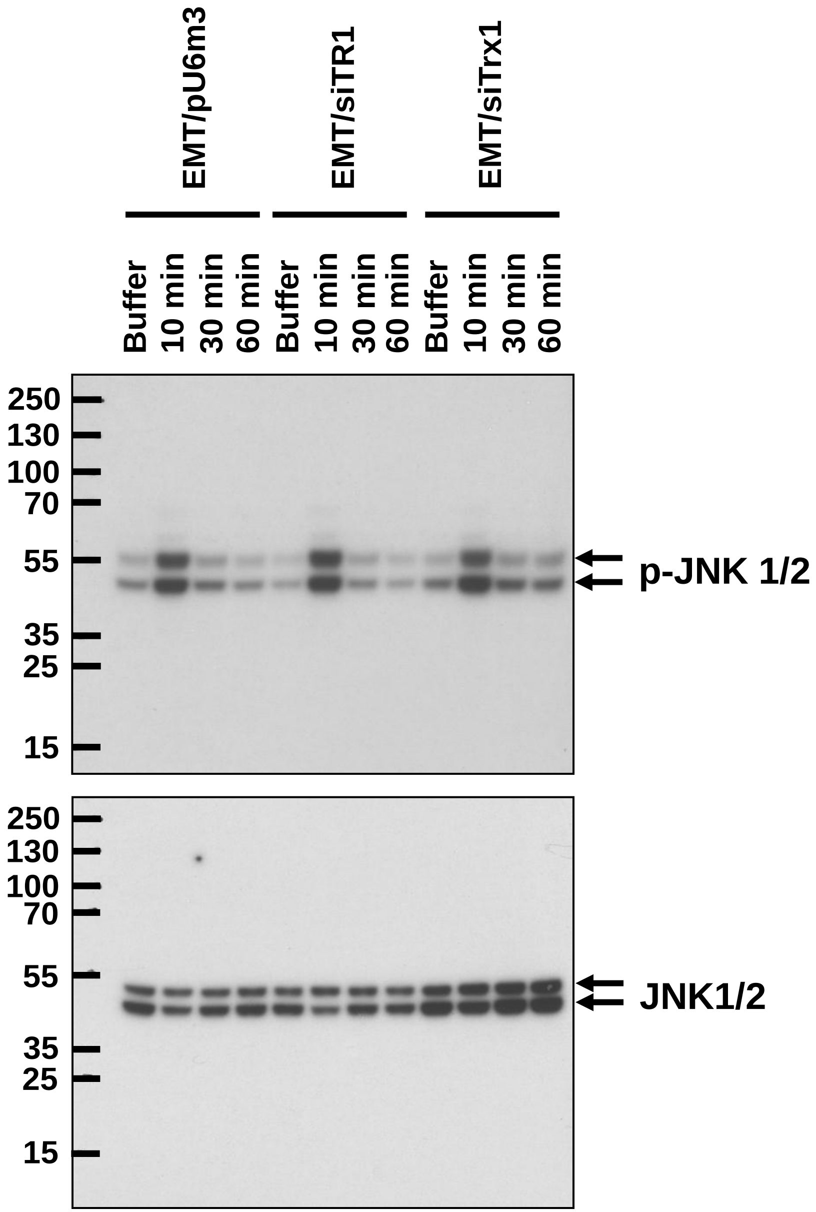

Figure 2A, lower panel

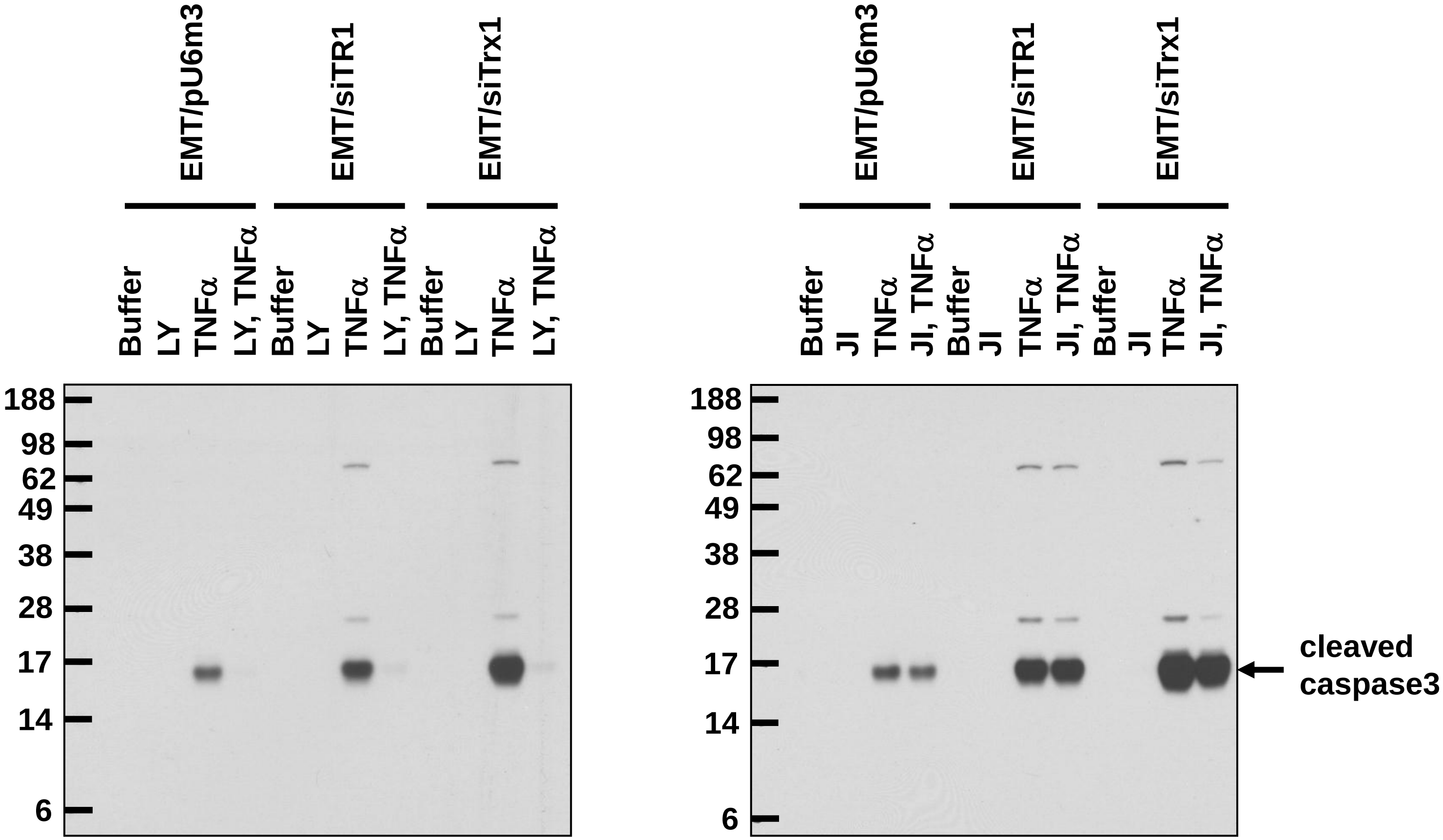

Figure 2B, upper and middle panel

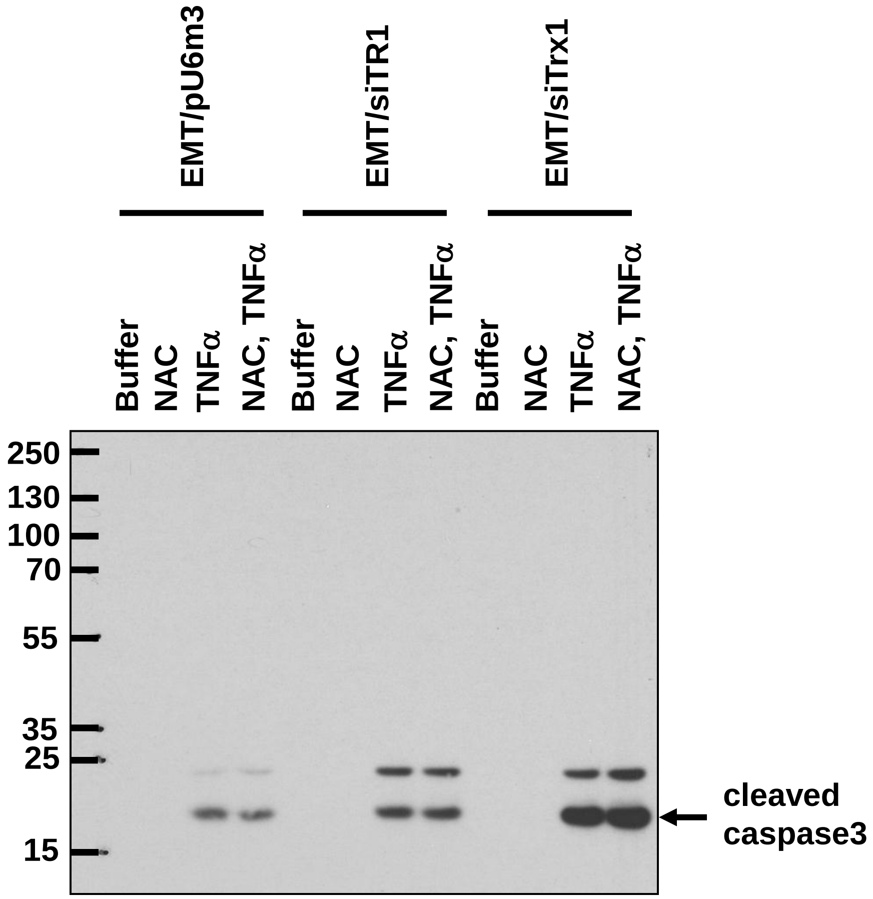

Figure 2B, lower panel

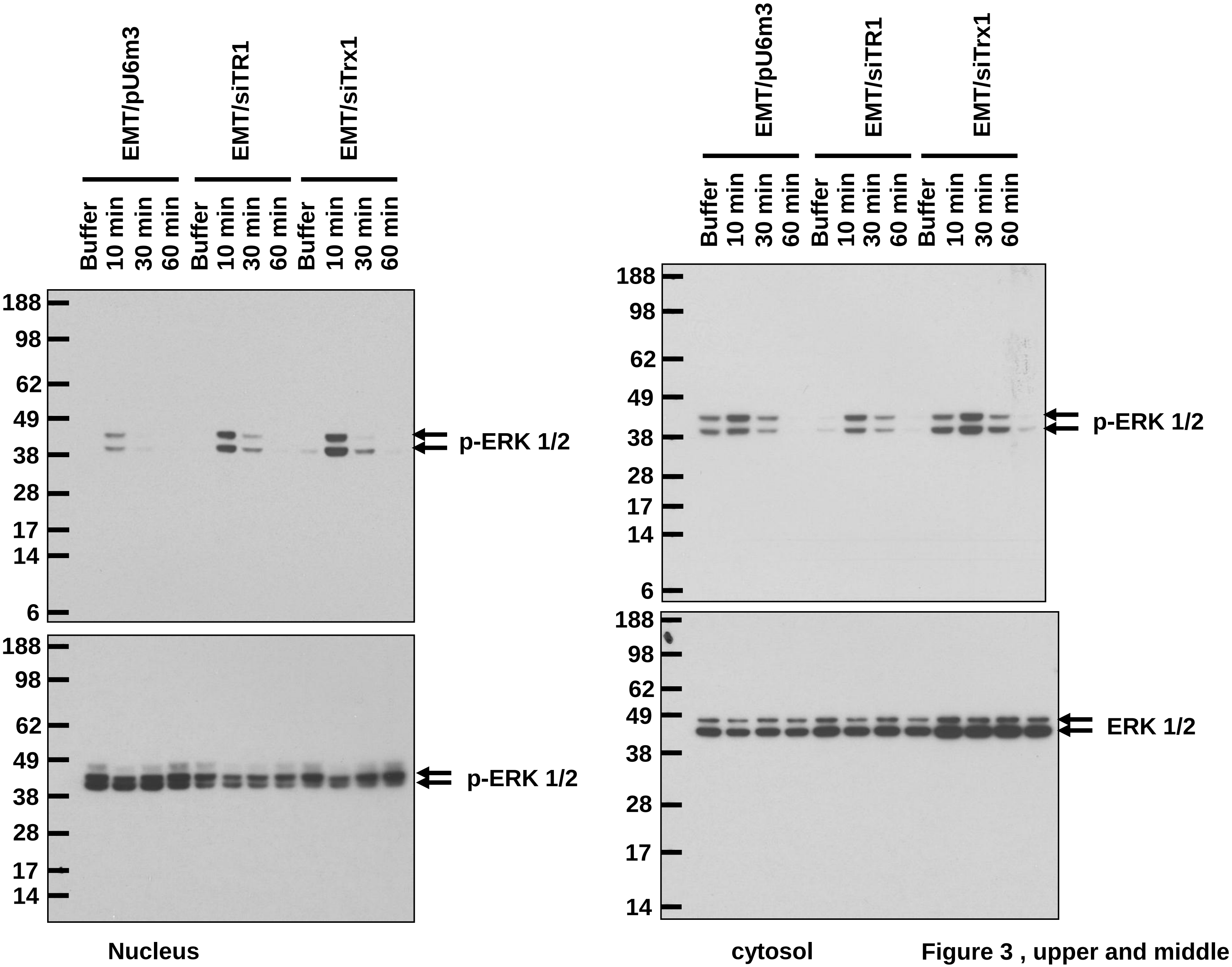

Figure 3 , upper and middle panel

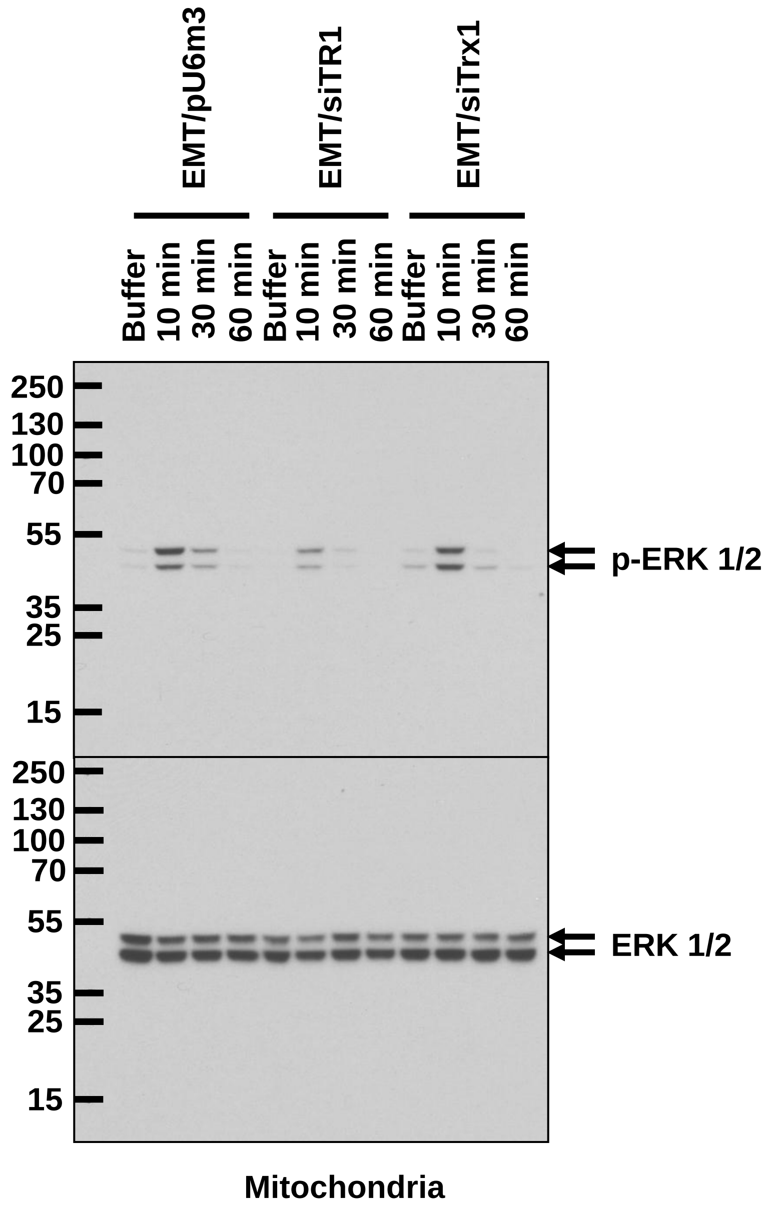

Figure 3, lower panel

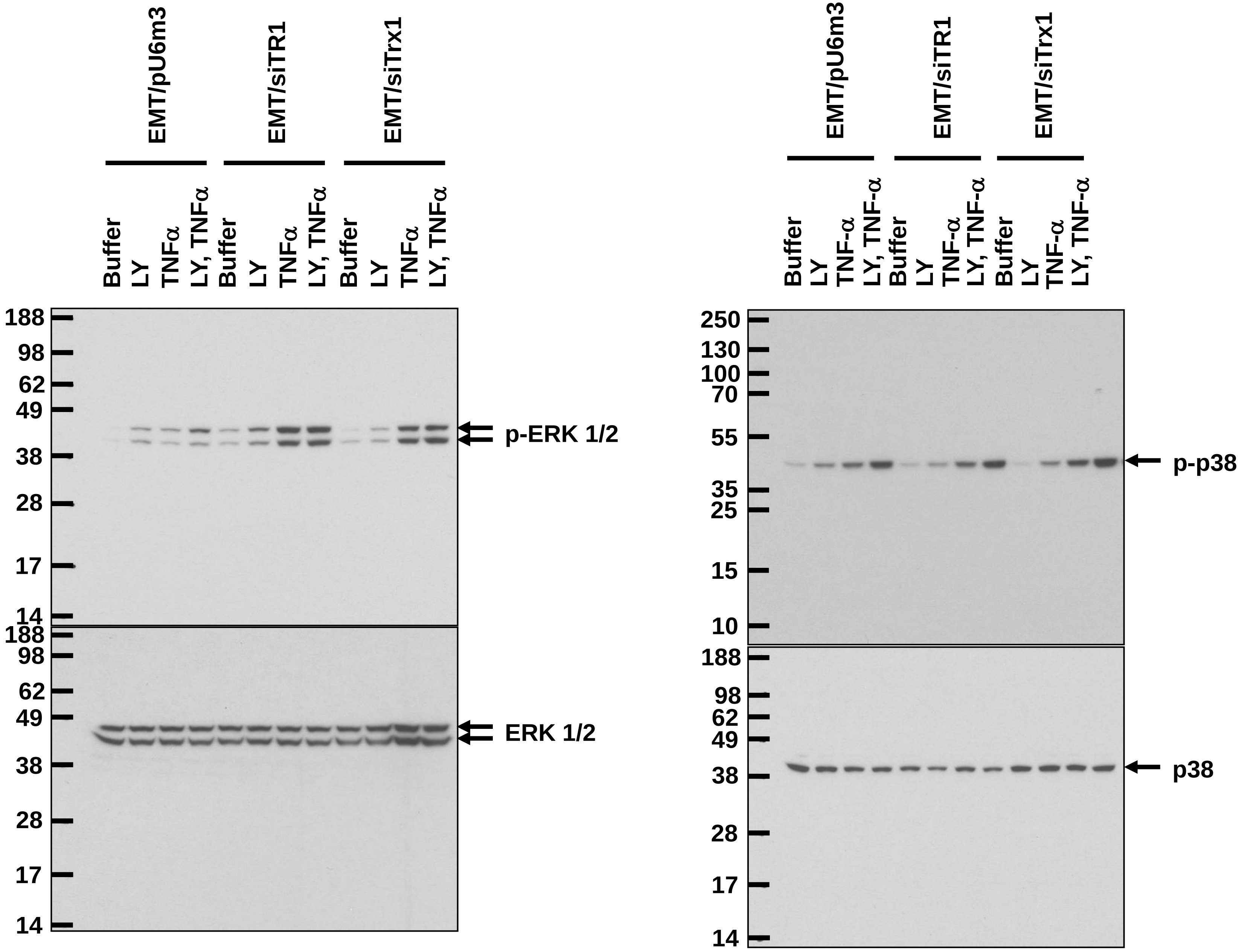

Figure 4A , upper and middle panel

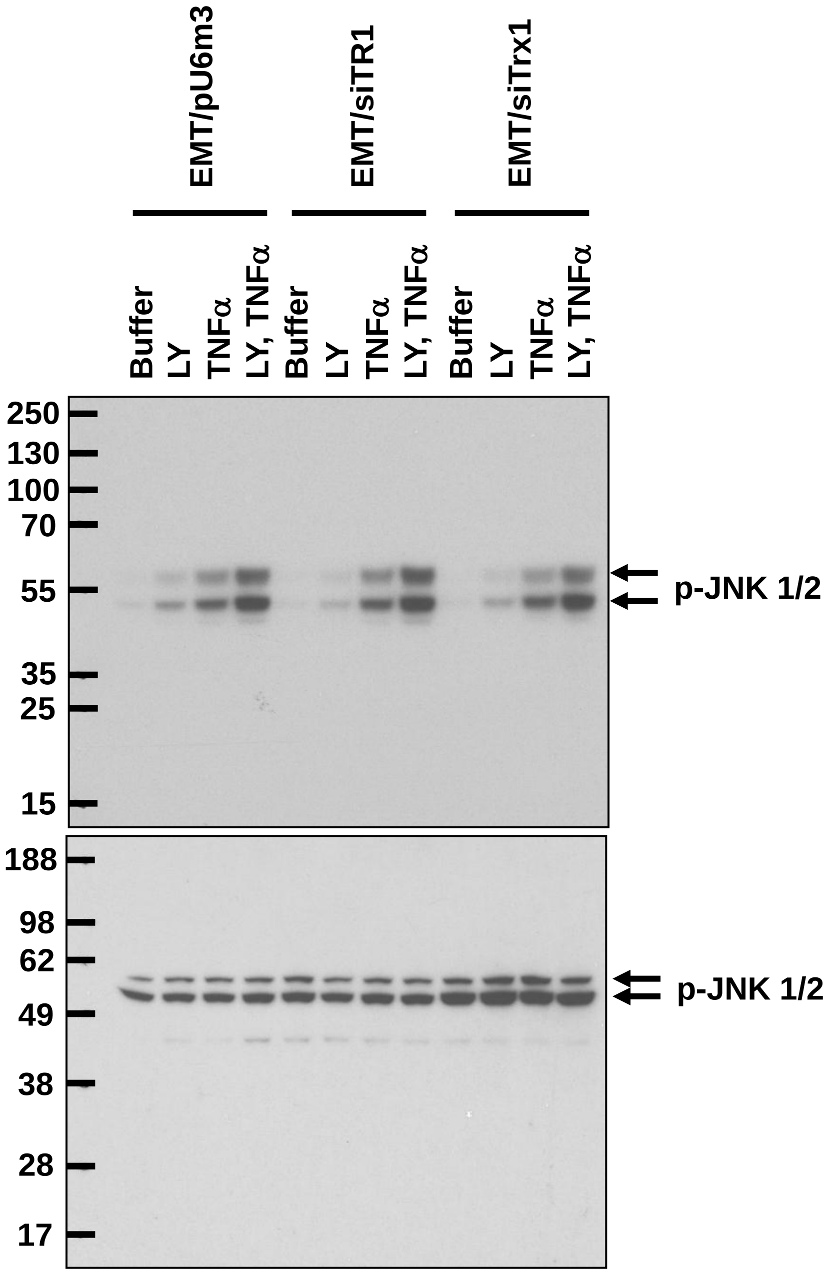

Figure 4A, lower panel

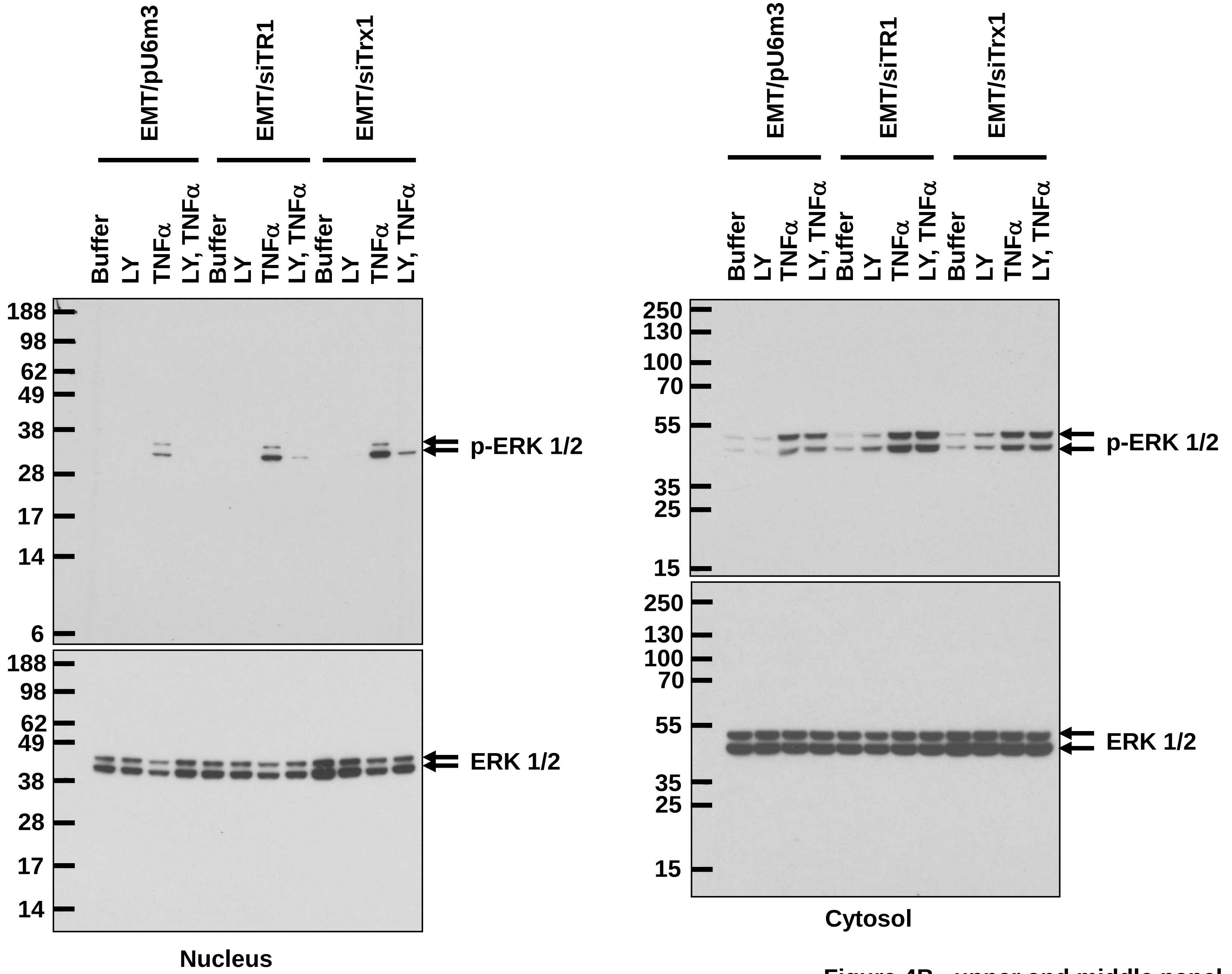

Figure 4B , upper and middle panel

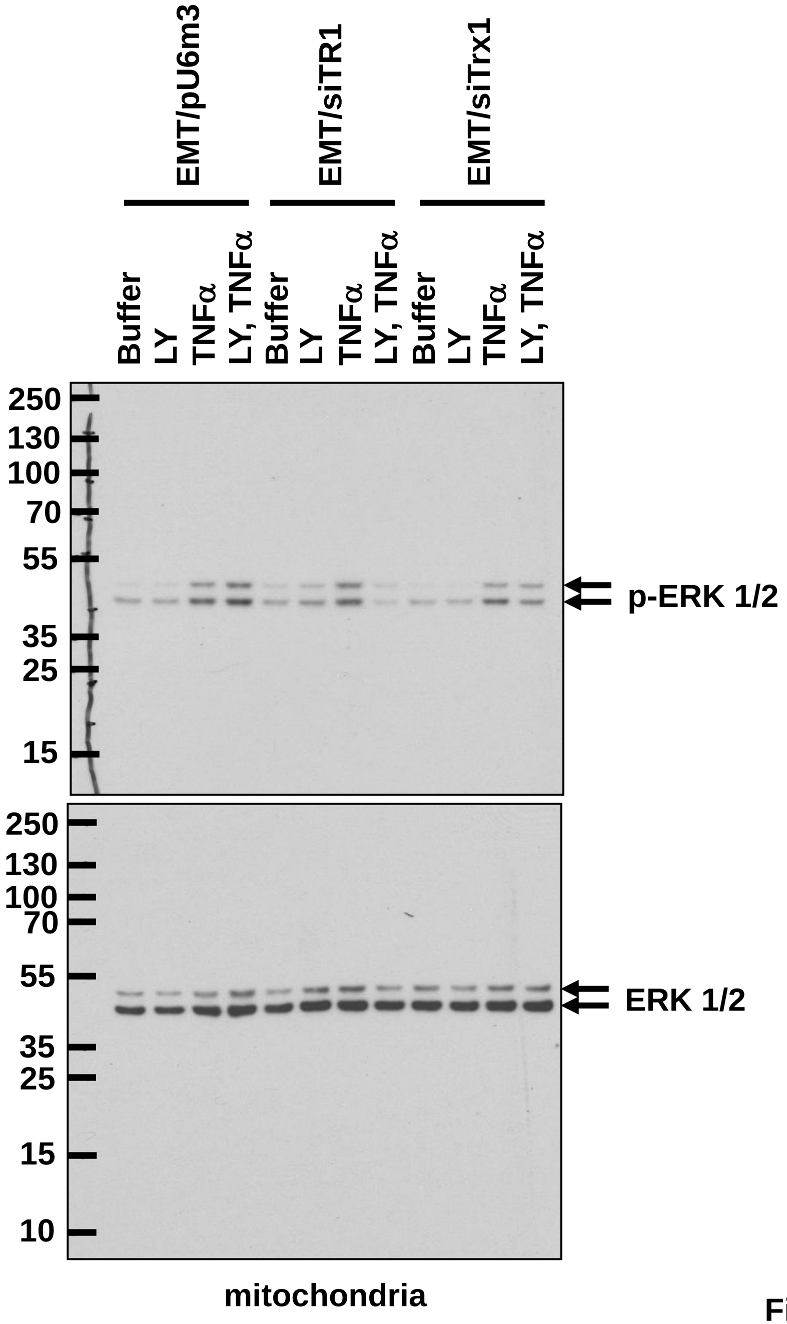

Figure 4B , lower panel
